# TumorHoPe: A Database of Tumor Homing Peptides

**DOI:** 10.1371/journal.pone.0035187

**Published:** 2012-04-16

**Authors:** Pallavi Kapoor, Harinder Singh, Ankur Gautam, Kumardeep Chaudhary, Rahul Kumar, Gajendra P. S. Raghava

**Affiliations:** Bioinformatics Centre, CSIR-Institute of Microbial Technology, Sector 39A, Chandigarh, India; University of South Florida, United States of America

## Abstract

**Background:**

Cancer is responsible for millions of immature deaths every year and is an economical burden on developing countries. One of the major challenges in the present era is to design drugs that can specifically target tumor cells not normal cells. In this context, tumor homing peptides have drawn much attention. These peptides are playing a vital role in delivering drugs in tumor tissues with high specificity. In order to provide service to scientific community, we have developed a database of tumor homing peptides called TumorHoPe.

**Description:**

TumorHoPe is a manually curated database of experimentally validated tumor homing peptides that specifically recognize tumor cells and tumor associated microenvironment, i.e., angiogenesis. These peptides were collected and compiled from published papers, patents and databases. Current release of TumorHoPe contains 744 peptides. Each entry provides comprehensive information of a peptide that includes its sequence, target tumor, target cell, techniques of identification, peptide receptor, etc. In addition, we have derived various types of information from these peptide sequences that include secondary/tertiary structure, amino acid composition, and physicochemical properties of peptides. Peptides in this database have been found to target different types of tumors that include breast, lung, prostate, melanoma, colon, etc. These peptides have some common motifs including RGD (Arg-Gly-Asp) and NGR (Asn-Gly-Arg) motifs, which specifically recognize tumor angiogenic markers. TumorHoPe has been integrated with many web-based tools like simple/complex search, database browsing and peptide mapping. These tools allow a user to search tumor homing peptides based on their amino acid composition, charge, polarity, hydrophobicity, etc.

**Conclusion:**

TumorHoPe is a unique database of its kind, which provides comprehensive information about experimentally validated tumor homing peptides and their target cells. This database will be very useful in designing peptide-based drugs and drug-delivery system. It is freely available at http://crdd.osdd.net/raghava/tumorhope/.

## Introduction

Cancer is one of the leading causes of immature deaths all over the world [1]. In 2008, more than 7 million deaths (around 13% of all deaths) occurred due to cancer and more new cancer cases are expected (http://www.scribd.com/doc/48974229/Global-Cancer-Statistics-2011) [2]. Both developed and developing countries are in the grip of this deadly disease. Despite tremendous progress in the field of cancer research, still we are unable to design effective and efficient anti-cancer therapy [3]. The current anti-cancer drugs are not selective and kill both tumor cells and normal cells. Therefore, designing drugs that specifically target cancer cells without affecting normal cells is one of the most challenging tasks for researchers. In order to overcome this limitation, various approaches/strategies/delivery systems have been developed. These approaches include the use of engineered antibodies, cell penetrating peptides, tumor homing peptides, and aptamers [4].

In the last few years, tumor homing peptides have gained recognition as a drug delivery vehicle. Due to advancement in phage display technology, a large number of peptides have been discovered, which specifically bind to tumor cells. These peptides have a strong affinity towards a specific receptor/marker that is often present in many tumors and tumor vasculature. In several cases, some of these receptors/markers are over expressed in tumors, in comparison to their expression in normal tissues [5]. These peptides can also recognize angiogenic/metastatic lesions, which may not be detectable using other traditional methods [6]. Since these peptides home to tumor site/vasculature through the circulation, they are often called as tumor homing/ targeting peptide. Most of the tumor homing peptides have been identified by phage display technique [7] and have shown good accumulation at the tumor site, when injected in mice models [8]. Drugs conjugated to such peptides, when administered, have shown more efficacies in mice models [9], and many peptide based drugs are already in clinical trials [10], [11]. In addition, these peptides have also been successfully used to deliver various imaging agent and inorganic nanoparticles [12]. Peptide based therapy has many advantages over the conventional anti-cancer chemotherapy. They are more specific and thus, exhibit lesser side effects [10]. In addition, due to direct delivery of the anti-cancer agent/drug to the malignant tissue, chemo-resistance of the cancer cells can be overcome or reduced [13].

Despite the huge therapeutic potential of tumor homing peptides, till date, there is no database or bioinformatics method for designing of effective tumor homing peptides. In order to facilitate scientific community, we have made a systematic attempt to collect and compile experimentally validated tumor homing peptides.

## Materials and Methods

### Data collection and compilation

Data for TumorHoPe database is manually collected from published papers, patents, and web sites by using a combination of keywords like tumor homing peptides and tumor targeting peptides (Pubmed: http://www.ncbi.nlm.nih.gov/pubmed/). Experimentally validated sequences were collected from published literature. Sequences were also collected from the patents which are available at websites of United States Patent and Trademark Office and World Intellectual Property Organization.

### Database Architecture and Web interface

**Figure 1:**
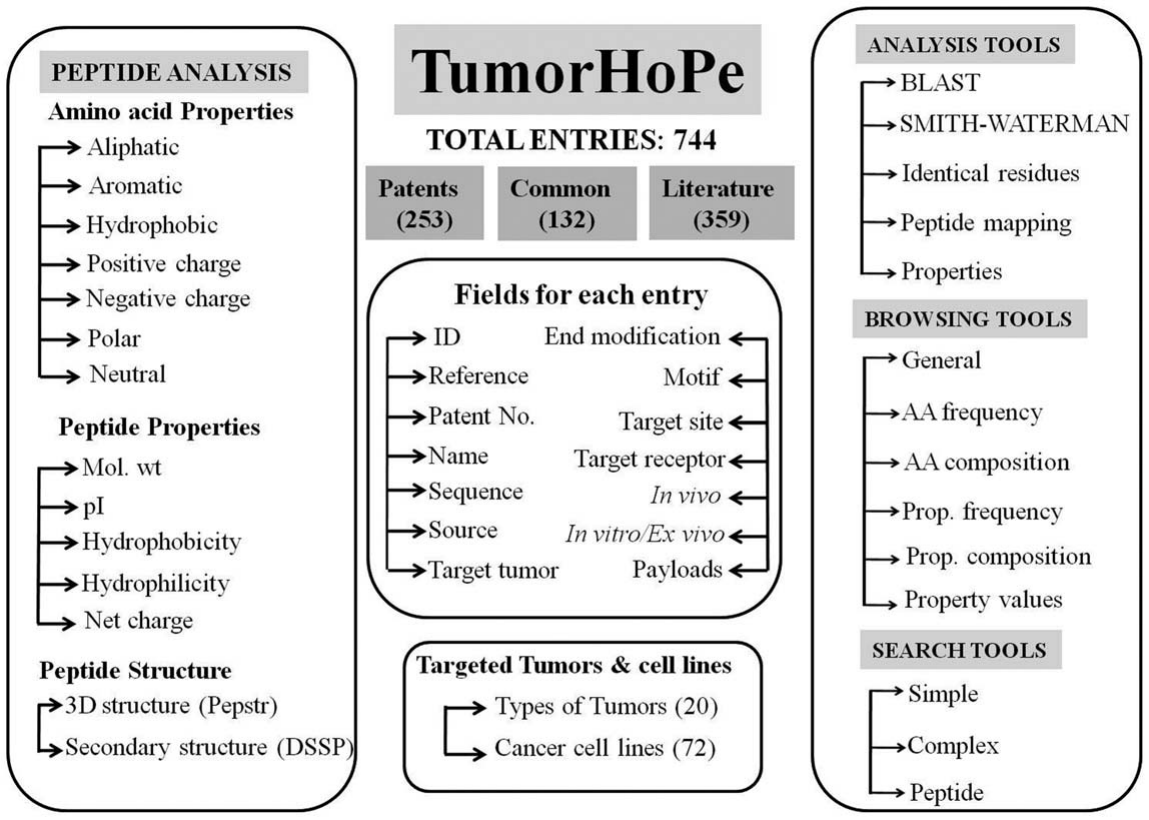
Overall architecture of TumorHoPe database.

TumorHoPe is built on Apache HTTP server 2.2 with MySQL server 5.1.47 as the back end and the PHP 5.2.9, HTML and JavaScript as the front end. Apache, MySQL and PHP are preferred as these are open-source softwares and platform independent. The architecture of TumorHoPe database is shown in Figure 1.

### Organization of data

TumorHoPe is a manually curated database which provides comprehensive information about peptides that target, bind to, and or home to tumor. Data for each peptide can be categorized as primary (*e.g.* peptide sequence, PubMed ID (PMID) and experimental details) and secondary data (*e.g.* secondary/tertiary structure, amino acid composition, frequency and physicochemical properties). Each peptide is assigned a unique entry number, and information is divided into different tables. Each table provides unique information.

#### Primary Data

Primary data contains general information of tumor homing peptides. All the information is extracted manually from various resources, mainly from publications and patents. As shown in the Figure 1, database provides comprehensive information on each peptide using more than 15 types of fields. Main fields are described as follows: (i) Sequence: it contains amino acid sequence of tumor homing peptide; (ii) Target tumor: it contains the name of tumor targeted by peptide; (iii) Target cell: it is type of cells targeted by peptide; (iv) Phage/library: It provides details of phage display library; (v) *in vitro*/*ex vivo:* it provides details of experiment carried out in *in vitro* and *ex vivo* conditions; (vi) Clone name: it provides information of clones of tumor homing peptides obtained by phage display technology against various tumors; (vii) Payloads: it provides information of conjugate attached with the peptide for imaging or drug delivery; (viii) Tumor homing/Tumor targeting peptides : it provides information whether peptide has been used *in vivo* experiment (tumor homing) or has been used *in vitro* experiments (tumor targeting).

#### Secondary Data

In the past, it has been shown that efficacy of tumor homing peptide depends on its structure [14]. Therefore, understanding of tertiary structure of these peptides is a prerequisite. Since these peptide structures are not available in Protein Data Bank (PDB) and existing methods for predicting tertiary structure of protein are unsuitable for predicting tertiary structure of peptides, we have predicted tertiary structure of tumor homing peptides using software PEPstr [15]. PEPstr is a state of art method for predicting structure of bioactive peptides. It is a *de-novo* method which builds a tertiary structure from predicted secondary structure and tight turns [15]. In addition, secondary structures of these peptides have also been predicted from predicted tertiary structure using software DSSP [16]. In our database, we maintain both secondary and tertiary structure of each peptide in PDB format.

In order to understand the nature of the peptide, it is imperative to know the constituents of each peptide. Therefore, in-house PERL scripts have been used for computing frequency and composition of each type of amino acid residue in all the peptides. This information is very useful to understand which residues/motifs are preferred in tumor homing peptides. Apart from amino acid frequency and composition, users may also want to know which types of residues are preferred like charged, polar, hydrophobic, etc. Therefore, we have computed frequency and composition of each class (aromatic, aliphatic, positively charged, negatively charged, neutral, polar and hydrophobic) of residues. This information is very useful to know which peptides are dominated by positively charged residues. We have also computed overall physicochemical properties (hydrophobicity, hydrophilicity molecular weight, iso-electric point and net charge) of each peptide [17], [18]. All above information is stored in the database as secondary data for analysis and browsing of peptides based on their properties.

### Implementation of tools

Apart from the collection of tumor homing peptides and their targets, a wide variety of information can be generated using the various online software/tools provided with TumorHoPe. Following are the main tools provided with the TumorHoPe database.

### Data searching

TumorHoPe is integrated with a user friendly interface for retrieving data from the database. A brief description of these interfaces is as follows:

#### Keyword Search

This interface allows users to search their query in most of the fields of database (*e.g.*, peptide sequence, motif, target tumor, target cell, source clone). One of the powerful features of this interface is that it allows users to select fields they wish to display in their results.

#### Complex Search

Advance search allows users to search the whole database in a stepwise manner. A query can be submitted using the following five steps: (i) fill the field name; (ii) choose any of four match operators ( = , >, <, and LIKE); (iii) fill the value, (iv) choose between two condition operators (AND & OR); and (v) press to add another query. Users are able to add multiple queries to search the database. In summary, advance search allows users to perform more complex queries in TumorHoPe.

#### Peptide Search

This interface is designed for searching a peptide sequence in the database. It allows two types of queries: (i) Containing peptide: it is for searching user defined peptide sequence in tumor homing peptides, and (ii) Exact search: it allows user to search tumor homing peptides, which are 100 percent identical to user's peptide.

### Browsing tools

We have designed powerful browsing facility that allows a user to browse data using various options. A brief description of interfaces designed for browsing are as follows:

#### Major Fields

**Figure 2:**
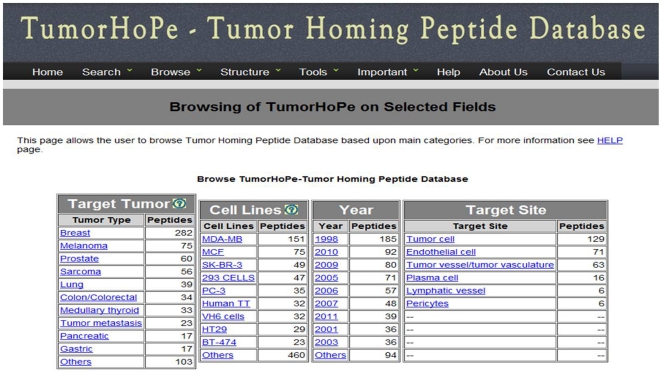
Screenshot of major fields page of TumorHoPe.

This interface allows a user to browse database on the following four major fields: (i) target tumor; (ii) cell lines; (iii) year of publication; and (iv) target site. As shown in Figure 2, it provides a number of peptides for different types of target tumor, target site and cell lines.

#### Amino Acid Frequency

This option is designed for searching or extracting peptides, which have desired frequency of specific type of residues. For example, user may search all peptides that have two Arg and three Ala. This is useful option of searching peptides dominated by particular type of residues.

#### Amino Acid Composition

This interface allows users to extract peptides from the database based on their amino acid composition. For example, user can search all the peptides that have all Arg residues by specifying 100% composition of Arg. User can easily extract tumor homing peptides with desired amino acid composition.

#### Physicochemical Property Frequency

User can extract tumor homing peptides with desired frequency of certain types of residues like, positively charged, negatively charged polar aliphatic, aromatic residues, etc. For example, user can easily extract all tumor homing peptides that have more than four positively charged residues.

#### Physicochemical Property Composition

This interface allows users to extract tumor homing peptides with desired composition of different types of residues like, positively charged, negatively charged, polar residues, etc. For example, user can easily extract all tumor homing peptides that have more than 80% positively charged residues.

#### Physicochemical Property Value

We have also computed physicochemical properties of each tumor homing peptide like hydrophobicity, hydrophilicity, net charge, iso-electric point. This tool allows users to extract tumor homing peptides with desired physicochemical properties. For example, user can search all peptides having net positive charge and molecular weight in the desired range [17], [18].

### Structure based browsing tools

In this database, predicted secondary and tertiary structure of each peptide has been stored. Three interfaces have been developed to extract structural information of tumor homing peptides. A brief description of these interfaces is as follows:

#### Secondary Structure (SS) composition

This interface allows users to browse peptides based on their secondary structure composition. We have assigned four types of secondary structure states (H-helix, E-beta strand, T-turn and C-coil) from DSSP. User can search tumor homing peptides with desired composition of these four types of secondary structure states. For example, user can search peptides that have more than 60% residues in the helix state.

#### Secondary Structure (SS) Search

This interface allows users to search segment of particular secondary structure states.

#### 3D structure

This interface is designed to browse tertiary structure of tumor homing peptides. It also allows user to search results on any field. First, it displays structure of peptide as static image. This image is clickable, and on click, it launches Jmol (http://www.jmol.org) after loading structure of peptides. This allows user to visualize 3D structure in any format or size/orientation supported by Jmol.

### Web-Based Tools

A number of web-based tools have been integrated in this database to facilitate further analysis of peptides. A brief description of these tools is as follows:

#### Blast Search

We have integrated BLAST search tool [19] that allows users to perform similarity based search against tumor homing peptides. This option allows users to submit one or more peptide sequences in FASTA format for performing BLAST search against tumor homing peptides.

#### Smith-Waterman Algorithm

In order to handle similarity search effectively in case of small peptides, we have integrated Smith-Waterman algorithm [20]. This option allows users to search tumor homing peptides in the database that are similar to their peptides. User can submit multiple peptide sequences in FASTA format.

#### Identical Residues

In addition to BLAST and Smith-Waterman algorithm, a simple algorithm has been integrated called Identical Residues. It takes user defined peptide sequence and aligns it with each tumor homing peptide using overlapping approach (alignment without gap). It displays query and target sequence with a number of identical residues in two peptides.

#### Peptide Mapping

It allows users to map tumor homing peptides on their peptide sequence. User may submit protein or polypeptide sequence on this page to identify segments that are identical to peptides in TumorHoPe.

## Results

TumorHoPe contains 744 peptides in which 359 peptides have been collected from research publications and 253 peptides have been collected from patents. The rest 132 peptides have been collected from both research publications and patents. As shown in Figure 2, peptides target more than 20 types of tumors. For example, 282 peptides target breast tumor, 75 peptides target melanomas, and 60 peptides target prostate tumor. In this study, we have covered more than 70 types of cancer cell lines. A few cell lines are found to be targeted by a large number of homing peptides. For example, 151 peptides target MDA-MB cell line, 75 peptides target MCF cell line, and 49 peptides target SK-BR-3 cell line (column 2 of Figure 2).

**Figure 3:**
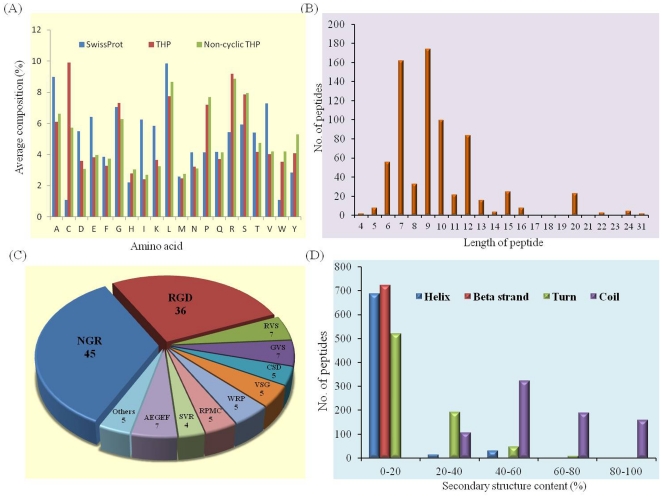
Distribution of tumor homing peptides in TumorHoPe. (A) Average amino acid composition of peptides (SwissProt proteins, tumor homing peptides (THP) and non-cyclic THP), (B) length wise distribution of tumor homing peptides, (C) distribution of peptides based on major sequence motifs, and (D) distribution of peptides based on secondary structure composition.

We have computed average amino acid composition of these peptides and observed that certain types of residues (Cys, Arg, Gly, Leu and Ser) are more abundant in tumor homing peptides (Figure 3A). We have also computed and plotted the average amino acid composition of equal number of proteins (744) extracted from SwissProt. As shown in Figure 3A, tumor homing peptides have relatively higher average composition of Arg, Cys and Trp compared to SwissProt sequences, while residues Gly and Leu are abundant in both the cases. As shown in Figure 3A, Cys residue has the highest average composition. This is unusual as composition of Cys residues is very low in natural proteins and peptides. We have further examined the homing peptides and found that a wide range of peptides are cyclic where start and end residues are cysteine. In order to avoid biasness in our analysis, we computed average amino acid composition of non-cyclic tumor homing peptides (cyclic peptides were removed). As shown in Figure 3A, the composition of Cys of non-cyclic peptides is lower than the composition of Cys in all tumor homing peptides, but still it is significantly higher than the composition of Cys in normal proteins (SwissProt proteins). In addition, composition of Arg is also found to be very high. This is in agreement with various studies, which showed that positively charged residues are usually preferred in homing peptides. We have also calculated the frequency of poly-Arg (RR, RRR and RRRR) in tumor homing peptides (data not shown). Only 30 peptides contain RR motif, while no peptide has RRR motif. Therefore, high average composition of Arg in tumor homing peptides is not due to the presence of poly-Arg peptides/motifs and it may be due to the presence of some common motifs like RGD, NGR, etc. which contain Arg. It is also observed that most of the peptides have 7, 9, 10 and 12 residues (Figure 3B). This could be due to the more frequent use of phage display libraries consisting of 7 mer/9 mer peptides.

In past, many tumor homing motifs have been identified from homing peptides. These are smallest peptides that can selectively recognize tumor cells and tumor vasculature. One of the common motifs is RGD, which has a tumor homing ability. RGD specifically recognizes integrin (αvβ3 and αvβ5) receptor, which is a good marker of angiogenic blood vessels [21]. This motif has been studied extensively and many tumor homing peptides have been designed by incorporating this motif. Similarly, NGR motif has also been identified from tumor homing peptides, which specifically bind to cells expressing aminopeptidase N, a membrane-bound metallopeptidase that plays important roles in tumor angiogenesis [22]. We have examined all such motifs present in tumor homing peptides in our database and computed the number of peptides containing each of the motifs. Distribution of peptides containing most frequent motifs is shown in Figure 3C. It is observed that 45 peptides contain NGR motif, while 36 peptides contain RGD motif. We have also examined other motifs that occur at least in four homing peptides.

We have computed secondary structure composition of each peptide and classified peptides based on secondary structure content. As shown in Figure 3D, most of the peptides have no beta-strand or less than 20% beta-strand. A similar trend is observed for helix. Only few peptides have 40–60% helix content. In case of turn, most of the peptides have less than 20% turns content and a sharp decrease in number of peptides having turns up to 60%, is observed. In case of coil, a large number of peptides have most of the residues forming coil. This is expected as it is difficult to maintain a regular structure in small peptides. This is just a primary analysis, and a detailed analysis is required in order to understand the relation between structure and tumor homing capability of the peptides.

## Discussion

TumorHoPe database is developed to serve the scientific community working in the area of peptide based cancer therapeutics. Although many peptide databases, *e.g.* MHCBN [23], Bcipep [24], HMRbase [25], MimotopeDB [26] , CAMP [27], and ANTIMIC [28] have been published, yet, at present, to the best of our knowledge, there is no database available that provides detailed information about the tumor homing peptides. Therefore, we have developed this database, which not only saves time and effort of researchers involved in the field, but will facilitate the biological discovery process. We have compiled 744 peptides with experimental and structural information, making use of literature and patents.

Although plethora of papers has been published on therapeutic potential of tumor homing or targeting peptide in anti-cancer drug delivery and development, results have not come up to the expectations. We hope that this collection of tumor homing peptides will be very helpful to researchers to design novel peptides, which can be used further for developing novel anti-cancer drugs. In addition, TumorHoPe will also be useful for the generation of prediction models for novel tumor homing peptides. The understanding of the role of amino acids of tumor homing peptides is important for their rational drug designing. We have integrated various analysis tools, which can be exploited by users to search and modify their peptides in order to enhance the specificity of the query peptides. Since most of the tumor homing peptides bind to particular receptors present on tumor cells, structure of these peptides may play an important role in their binding to their receptors. Therefore, we have predicted 3D structures of all the peptides. Users can exploit this information for docking or molecular dynamics of the peptide-receptor complex. We anticipate that this thorough and comprehensive database will be extended to effective completeness and then maintained and its content expanded, with constantly enhanced search and analysis features.

### Submission and Update of TumorHoPe

The online data submission tool allows a user to submit a newly identified tumor homing peptide in the TumorHoPe database. However, before including in TumorHoPe, we will confirm the validity of new entry in order to maintain the quality. Our team is also searching and adding new entries of tumor homing peptides from published literature. In order to maintain the consistency, we will revive the TumorHoPe database.

### Availability and Requirements

TumorHoPe is freely available at http://crdd.osdd.net/raghava/tumorhope
